# Towards evidence-based computational statistics: lessons from clinical research on the role and design of real-data benchmark studies

**DOI:** 10.1186/s12874-017-0417-2

**Published:** 2017-09-09

**Authors:** Anne-Laure Boulesteix, Rory Wilson, Alexander Hapfelmeier

**Affiliations:** 1 Institute for Medical Information Processing, Biometry and Epidemiology, LMU Munich, Marchioninistr. 15, Munich, 81377 Germany; 2 Institute of Medical Statistics and Epidemiology, Technical University Munich, Ismaninger Str. 22, Munich, 81675 Germany

**Keywords:** Method evaluation, Good practice, Comparison study, Clinical trial

## Abstract

**Background:**

The goal of medical research is to develop interventions that are in some sense superior, with respect to patient outcome, to interventions currently in use. Similarly, the goal of research in methodological computational statistics is to develop data analysis tools that are themselves superior to the existing tools. The methodology of the evaluation of medical interventions continues to be discussed extensively in the literature and it is now well accepted that medicine should be at least partly “evidence-based”. Although we statisticians are convinced of the importance of unbiased, well-thought-out study designs and evidence-based approaches in the context of clinical research, we tend to ignore these principles when designing our own studies for evaluating statistical methods in the context of our methodological research.

**Main message:**

In this paper, we draw an analogy between clinical trials and real-data-based benchmarking experiments in methodological statistical science, with datasets playing the role of patients and methods playing the role of medical interventions. Through this analogy, we suggest directions for improvement in the design and interpretation of studies which use real data to evaluate statistical methods, in particular with respect to dataset inclusion criteria and the reduction of various forms of bias. More generally, we discuss the concept of “evidence-based” statistical research, its limitations and its impact on the design and interpretation of real-data-based benchmark experiments.

**Conclusion:**

We suggest that benchmark studies—a method of assessment of statistical methods using real-world datasets—might benefit from adopting (some) concepts from evidence-based medicine towards the goal of more evidence-based statistical research.

## Background

The role of a medical practitioner is to perform interventions on patients that are as beneficial as possible in a broad sense, taking into account both the short term and the long term, health outcomes, quality of life and often other aspects, such as ease of use or extended application to a wider set of indications. In contrast, it is the goal of medical researchers to *develop* new interventions that are superior—or non-inferior with fewer side effects—to those already in existence. Rightfully, the unbiased systematic evaluation of the new and previously existing interventions is considered crucial by the medical community and is a focal point of medical literature. The concept of “evidence-based medicine” (EBM) has been receiving growing attention and credibility for decades.

We can draw a parallel between medicine and computational statistics, with the statistical consultant analogous to the medical practitioner and the applied statistical researcher to the medical researcher. See Table 1 for an overview of this analogy that will be developed throughout the paper. The role of a statistical consultant is to analyze the client’s data such that the results help to answer a research question as completely as possible, uncovering and approximating a truth that is assumed to exist behind this question. Once more, there may be other considerations, such as cost and computation time. The goal of applied statistical researchers is to *develop* data analysis methods and tools that are, again, in some sense superior to those already in existence. However, here the parallel between medicine and computational statistics ends, as the unbiased evaluation of these new statistical methods and tools in real-data settings, including their comparison to existing methods, is given usually only poor attention in the literature. In this paper, we explore the disparity between evidence-based medicine and “evidence-based computational statistics” by examining the state of methodological aspects of benchmark studies, the systematic comparison of statistical methods using real datasets.

**Table 1:** Analogy between clinical research and computational statistical research

	Clinical research	Computational statistical research
Trial type	In vitro/animal study	Simulation
	Clinical trial	Benchmark study
	Blinded	Neutral and blind analysis
	(Placebo) controlled	(Null-model) controlled
	Cross-over	Paired samples
	Multi-arm	Multiple methods
Investigators	Trialist	Researcher conducting benchmark experiment
	Medical researcher	Methodological researcher in computational statistics
	Sponsor	Methodological researcher in computational statistics
Observation unit	Clinical trial patients	Real datasets
Comparators	Therapies, interventions and controls	Statistical and machine learning methods
Problem	Treatment of medical condition	Answering a question using data, e.g. prediction problem
Context	Patient’s preference, social context	Substantive context
	Personalized medicine	Meta-learning
Objectives	Improving patient’s health	Yielding reliable answer, e.g. increasing prediction performance
	Selecting and applying therapy to patient	Selecting and applying methods to datasets
	Application by medical practitioner	Application by statistical practitioner/consultant
Endpoints	Relevant clinical endpoints	Error rate, AUC, computing time, etc.
	Missing value (e.g. dropout)	Failure to produce output

Greenhalgh et al. [1] state: “It is more than 20 years since the evidence based medicine working group announced a “new paradigm” for teaching and practicing clinical medicine. Tradition, anecdote, and theoretical reasoning from basic sciences would be replaced by evidence from high quality randomized controlled trials and observational studies, in combination with clinical expertise and the needs and wishes of patients.” Our aim is to start a discussion on “evidence-based” data analysis in which “tradition, anecdote, and theoretical reasoning from basic sciences [including simulations] would be [complemented] by evidence from high-quality [benchmark studies], in combination with [statistical] expertise and the needs and wishes of the [substantive scientists]”. Our discussion is based on an analogy between clinical trials, which play a crucial role in evidence-based medicine, and real-data-based benchmarking experiments in methodological statistical science, with datasets playing the role of patients and methods playing the role of medical interventions (see Table 1).

In computational statistics, “evidence” can be generated through theoretical considerations (e.g., the proof that an algorithm converges or the asymptotic relative efficiency of a test), by simulations (i.e., with artificial datasets randomly drawn from a particular distribution) or through real-data examples. However, theory is often of little help in highly complex real-world situations, since it usually requires unrealistic simplifying assumptions regarding the data structure. In this paper we focus on the role of real-data analysis (as opposed to simulations) and the design of such studies. This type of evidence can be seen as “empirical evidence”.

In a specific example of the greater incorporation of evidence in evaluating and presenting statistical methodology, the recently established STRATOS (STRengthening Analytical Thinking for Observational Studies) cooperation [2] aims at providing guidance regarding the choice of statistical method based on empirical evidence and experts’ experience in the context of observational studies in medical research. However, such groundbreaking projects are still in their infancy and the concept of evidence and the role of real data in this context are not yet well defined.

In machine learning—a field focused on *prediction* as opposed to *explanation*—ideas relating to evidence from real data are becoming commonplace. Systematic comparison studies, often denoted *benchmark experiments*, based on real data are a core of the literature, their realizations made substantially easier through the use of databases of datasets available for benchmarking, such as the UC Irvine (UCI) machine learning repository [3] and the OpenML platform [4]. Machine learning challenges [5], which can be seen as collective benchmarking studies, are also receiving substantial attention from the community. Machine learning scientists work to obtain empirical evidence on the performance of algorithms (often algorithms to construct prediction models) on real datasets in analogy to medical doctors obtaining empirical evidence on the performance of therapies on human patients (see Table 1). Machine learning scientists are further aware of the no-free-lunch theorem (i.e., that no algorithm works best in all situations) and again address this problem through evidence-based research, by evaluating which algorithm performs best in which situations and even automatizing this process—a task known as meta-learning.

We statisticians are in general reluctant to adopt the concept of evidence-based decision-making: that the choice of statistical method to use in a given situation should be guided primarily by “evidence” in general and empirical evidence more specifically. This reluctance is also strong in the context of prediction modelling, which lies in the domains of both statistics and machine learning and has long been addressed by machine learners in benchmark experiments. Some statisticians feel a more evidence-based approach implies the jettisoning of the experience of the statistical consultant in favor of a suspect set of guidelines inspired by oversimplification. The idea that the choice of a method may be reached in a more or less automatic manner makes us feel unwell. Statisticians often argue that no ruleset or guideline can replace the judgement of an expert statistician, nor can a ruleset take into account all aspects of a problem, such as the substantive context. Interestingly, these are exactly the types of arguments invoked by EBM-sceptics. Medical doctors questioning EBM argue that an evidence-based approach based on systematic rules cannot cope with the complexity of individual cases—e.g., with respect to multi-morbidity—and, again, ignores important considerations, such as the wishes and social backgrounds of patients.

The existence of specific datasets (in statistics) or patients (in medicine) with complexity that cannot be accommodated by simple evidence-based rules may be seen as an argument in favor of the need for more evidence, i.e. evidence tailored to particular dataset or patient profiles. This need has long been acknowledged in medical research and is being addressed in the emergence of personalized/individualized medicine, with subgroup and interaction effect analyses in clinical trials being simple steps in this direction. Similarly, in computational sciences, the development and use of meta-learning are steps towards tailored algorithms.

Medical doctors or statisticians may still maintain that even the best and most individualized evidence cannot replace expert intuition, nor adequately take into account the specific context of the choice. This is a controversial issue. One may argue that an expert’s intuition is simply the result of the unconscious collection of evidence from personal experience during a career, and that such evidence could also be formalized as systematic rules. If so, the question becomes whether one can trust machines to derive rules as reliably as the brain of an expert, and whether this can be achieved in practice considering the current state-of-the-art of computational sciences. In a given situation, the amount of information (e.g., number of cases) and the source of the information (e.g., type and quality of studies and data) play further crucial roles in this appraisal.

Statisticians may also contend that the need for empirical evidence in statistics is not as strong as in medicine, as theoretically one can subject a dataset to as many statistical methods as one desires, while the same cannot be said for patients and interventions. Clearly, it does not harm a dataset to undergo different statistical analyses, but it may harm patients to undergo different interventions before identifying the most appropriate. While the sense of this argument is evident, it is well known that the approach of performing a large number of statistical methods on a dataset and deciding which one is the “right one” based on the results may yield substantial problems relating to the idea of “fishing for significance” [6]. An illustration is provided in an experiment in which they asked 29 statisticians to analyze a dataset with the goal of assessing the potential correlation between skin color of football players and red cards [7]. Perhaps surprisingly to some, but likely not to many statisticians, the researchers obtained very different—and partly contradictory—answers! Which result should be reported as definitive? Researchers are obviously tempted to report that which is most fitting to their goals. Due to multiple comparison effects, this strategy is likely to yield false research findings that are simply the result of optimization and data dredging and would fail to be validated using independent data [6, 8].

When considering types of evidence, statisticians are usually keener to evaluate their methods using data simulated from known distributions as opposed to conducting benchmark studies consisting of a large number of real datasets. The use of simulations as opposed to real-data analysis can be considered analogous to using in vitro studies or animal trials as opposed to patient-based experiments: one can control important factors—genotypes, age, diet, etc. in medicine; the dataset size, the signal strength, etc. in computational science—and thus obtain homogeneous groups and “know the truth”. In the context of, say, hypothesis testing or the estimation of a variable’s effect for the purpose of explanation (as opposed to prediction), the truth is unknown in real data, thus making simulations indispensable for evaluating how well statistical methods uncover the truth. Moreover, one can simulate as much data as computationally feasible, allowing reliable systematic evaluation of the methods in the considered simulation settings. In many situations, simulations are indispensable. However, even with the best simulations, one would often remain uncertain as to the performance of the examined methods in the much more complex real world.

In this context, we would like to start and fuel a discussion on the appropriate design of real-data studies yielding evidence in statistical research, always with careful consideration of a dataset’s specificities/substantive context and without discarding expert intuition and simulations. In analogy to EBM and the choice of therapies, large-scale benchmarking research in statistics may yield tentative rulesets and guidelines to facilitate the choice of data analysis methods *without dictating* them. We would like to discuss the question—without claiming to have the ultimate answers—of the role of EBM-inspired concepts in real-data benchmark analysis in computational applied statistics.

In this paper, we assume that the performance of a statistical method on a real dataset can be objectively assessed using some criterion. This is the case, for example, for prediction methods: natural criteria are error measures such as the error rate (in the case of binary classification) or the Brier score (in the case of survival prediction), which can be estimated through the use of resampling techniques such as cross-validation. This is the context we will use to explain our ideas. Methods whose performance on real datasets cannot be quantified using real data, such as hypothesis tests or effect estimation procedures, are not considered here. Moreover, unless stated otherwise we assume that a method is well defined and runs automatically on a dataset without human intervention such as parameter initialization or preprocessing. The issue of human intervention is discussed further in “Role of the user” section.

While one cannot incorporate all aspects of EBM into the context of the evaluation of statistical methods using benchmarking, we claim that some precepts commonly accepted in EBM may be helpful in defining a concept of “evidence-based computational science”. A simple example is that of sample size, an extensively researched question on the number of patients required in a clinical trial in order to make valid statistical claims on any result. Analogously, in benchmarking, in order to draw conclusions from real-data analysis beyond illustrative anecdotic statements, it is important to have considered an adequate number of datasets; see Boulesteix et al. [9] for a discussion on the precise meaning of “an adequate number”. In the remainder of this paper, we discuss further concepts essential to formulating evidence-based statements in computational research using real datasets. After the presentation of a motivating example in “Motivating example” section, the significant question of the definition of selection criteria for datasets is addressed in “Selecting datasets: a major challenge” section, while other concepts from medical sciences such as analysis protocols, placebos, evidence levels, and bias are discussed in “Further EBM-related concepts” section (see Table 2 for an overview of these concepts).

**Table 2:** Some ideas for the improvement of benchmarking practice

Clinical research	Treated in	Transfer into computational	Example(s)
		statistical research?	
Sample size calculation	[9]	Possible and desirable [9]	[35]
Strict inclusion criteria	Sec. 3	Possible and desirable	[20, 21, 35]
Trial protocol	Sec. 4.1	Principle might be helpful in adapted form	
Quality control	Sec. 4.2	Principle might be helpful in adapted form	
		e.g. via platforms like OpenML [4]	
Placebo/reference	Sec. 4.3	Principle might be helpful in adapted form	
Blinding	Sec. 4.4.1	Principle might be helpful in adapted form	
Intention-to-treat	Sec. 4.4.2	Adequate treatment and reporting of	[29]
		missing values: possible and desirable	
Levels of evidence	Sec. 4.5	Principle might be helpful in adapted form	

## Motivating example

Since the early 2000s, numerous supervised classification algorithms have been proposed in the bioinformatics and statistics literature to handle the so-called *n*≪*p* problem, i.e. the case where the number *p* of candidate covariates (for example, gene expression data collected using microarray technology) by far exceeds the number *n* of patients in the dataset. Common approaches to handle this dimensionality problem include preliminary variable selection, dimension reduction, penalized regression or methods borrowed from machine learning [10].

In this setting, the comparison of existing classification methods using real microarray datasets has been the topic of a number of papers [11–18], which do not aim at demonstrating the superiority of a new method proposed by the authors: they consider only existing methods, thus implying a certain level of neutrality [19]. Most of these papers consider measures such as the error rate or the area under the curve to assess the performances of the classification methods. For each considered performance measure, the results are essentially presented in the paper as an *N*×*M* table, where *N* is the number of considered real datasets and *M* is the number of considered methods. The number *M* of methods varies across papers and ranges between *M*=2 and *M*=9. These methods are sometimes combined with different variable selection or dimension reduction techniques, thus yielding a higher number of investigated method variants. Considered methods vary across papers and include, for example, discriminant analysis, tree-based methods, nearest neighbors, or support vector machines.

We feel that these studies contain major flaws if examined in the light of the principles of clinical research. First of all, most are underpowered: with the exception of the study by de Souza et al. [18] (*N*=65 datasets) and that of Statnikov et al. [17] (*N*=22 datasets), they use between *N*=2 and *N*=11 datasets to compare the methods, too few to achieve reasonable power when comparing the performances of classification methods [9]. Following the sample size calculation approach outlined in Boulesteix et al. [9], the required number of datasets to detect a difference in error rates between two methods of, say, 3% with a paired sample t-test at a significance level of 0.05 and a power of 80% is as high as *N*=43 if the standard deviation (over the datasets) of the difference in error rates is 7%—a standard deviation common in this setting [9].

Furthermore, it is not clear how the datasets included in the studies were selected. No clear search strategy or inclusion criteria are described in the papers. Some of the datasets are used in several studies and sometimes denoted “benchmark datasets”, but none are used in all studies. The problem of the selection of datasets for inclusion in a benchmark study will be further discussed in “Selecting datasets: a major challenge” section. It is further unclear whether datasets have been eliminated from the study after having been originally included, since this task is not documented. We will come back to this problem in “Non-compliance and missing values” section.

Regarding neutrality, even if the aims of these studies are not to demonstrate the superiority of a particular “favorite” new method, authors are sometimes likely to have preferences for or better competence in one or several methods compared to the other(s). For example, one of the authors’ team may have a very strong background in a particular classification method, support vector machines, as suggested by their publication records. They are then likely to be more proficient users of this method than of the other(s) (although we can admittedly not verify this conjecture!), which may induce bias, as further discussed in “Neutrality and blinding” section.

## Selecting datasets: a major challenge

### The difficulty in sampling datasets

In the context of a clinical trial the population of interest is the population of patients with (or at risk for) a particular health condition who may benefit from the considered therapies. Diseased patients usually seek medical help, making it possible to draw from this population. For non-diseased patients (who may benefit from interventions such as prevention programs), representative sampling methods exist. On the whole, even if sampling is often a challenge and there remains the risk of bias, it is normally feasible to obtain reasonably representative samples from the population of interest.

In the context of benchmarking, the “population of interest” is the population of datasets in the focused research field whose structure and properties would allow them to be the target of the considered data analysis methods. In practice there are no simple sampling procedures for this population for a variety of reasons. Firstly, datasets are not typically systematically registered, except perhaps in specific fields (for example, most microarray datasets as considered in our illustrative example in “Motivating example” section are nowadays made publicly available via dedicated platforms). Secondly, the different approaches to access datasets (repositories, software packages, additional files or companion websites of published papers, personal contact with data owner) are all selective: they do not allow the drawing of independent datasets from the population of datasets. Thirdly, a “dataset” is not as well defined as a “patient” because it may be split, merged, preprocessed, etc. This is often the case in the context of microarray studies such as those considered in “Motivating example” section. These problems must be kept in mind when statistically interpreting the results of benchmarking, results which should be seen as conditional—given the data source (e.g., a particular data repository)—rather than as proper inference for a population.

Beyond these issues related to the sampling of datasets, an additional difficulty is related to the knowledge of the researcher conducting the benchmark experiment regarding the substantive context of the datasets. In some cases a profound knowledge of the substantive context is particularly important in conducting correct statistical analyses. One may then want to collect only datasets which are particularly well documented or datasets from one’s field of interest, an additional constraint which complicates the sampling procedure, reduces the number of potential datasets and may introduce a bias.

### Which patients to analyze in clinical trials, which datasets to analyze in benchmarking studies: a short overview of the common handling of inclusion/exclusion criteria

For clinical trials, strict criteria are applied when selecting patients to include in the study, with all patients fulfilling these criteria enrolled until a predefined sample size is reached. In contrast, for benchmarking studies in computational literature the criteria used for selecting datasets are most often non-transparent, with a few exceptions in the field of statistics for microarray data such as those considered in “Motivating example” section [20] and machine learning [21]. The consent of the data owner to make a dataset available is necessary but is often given informally. The minimum number of datasets to be included is usually not determined. Though conscious attempts to misrepresent the results (“cheating”) are probably rare, questionable elimination of datasets may be performed by honest researchers based on *a posteriori*, i.e. after seeing the results, plausible explanations such as, “the method did not work well on this dataset because it has property X, so it is justified to exclude it”, although this property—now presented as important—was not identified as an exclusion criterion beforehand. Such *a posteriori* elimination may lead to substantially biased results [22].

A related concern in the clinical field is the potential for the subsequent elimination from the study or change in treatment of a once-enrolled patient, a topic taken very seriously from a quality and analysis point of view and one highly studied in the literature. The handling of patients enrolled but failing to comply to treatment and the analogous situation of datasets producing nonsensical or missing results in real-data benchmark studies is discussed in “Non-compliance and missing values” section.

### Adopting principles from clinical research into benchmarking

We claim that strictly defined inclusion criteria could be applied when selecting datasets for inclusion in a benchmark study and that reasons for post-hoc exclusion should be reported thoroughly, for example, using flowcharts in the spirit of the CONSORT statement [23], a guide on the transparent reporting of trials. Inclusion criteria for datasets may be, for example, “the number of observations lies within a given range”, “the number of covariates lies within a given range”, “the scales of the covariates are of a certain kind”, “the outcome is applicable to the analysis of interest”—regression, classification, time-to-event, etc.—or requirements on the number of missing values.

There are three primary motivations for defining inclusion criteria, i.e. excluding datasets. Firstly, one typically excludes datasets which would render the assessment of performance of the statistical method difficult: for example, one may exclude small datasets because error estimation (e.g., with cross-validation) would be highly variable (a common issue in the context of the gene expression data considered in “Motivating example” section), or large datasets because the analysis would be too computationally demanding. This is similar to, for example, the exclusion of incontinent patients from a clinical trial if the outcome of interest is the result of a 24 h urine test. Secondly, a dataset may be excluded due to low data quality, for example, if the number of evident input data errors and the number of missing values are too great to yield proper analysis. Thirdly, one excludes datasets to focus on a particular setting and reduce heterogeneity for easier interpretation of the results; for example, in the context of gene expression one may decide to include only datasets measured with a particular type of microarray. These reasons for the exclusion of datasets contribute to the conditional character of results emerging from a benchmark study, as exclusion criteria do to the results of clinical trials. In both settings, there is a strong need for the precise reporting of inclusion criteria to enable fair appraisal of the corresponding findings.

A fourth reason—tightly related to the third—particularly affects benchmark studies undertaken in articles which are introducing a new statistical method: authors may exclude datasets because they have characteristics that are expected to lead to the demonstration of the inferiority of the new method. This exclusion of datasets may be a result of fear of *publication bias*, a topic of abundant literature in medical sciences, and also of concern in computational sciences [24]. The researchers may rightly worry that studies suggesting better performance of a new method are more likely to get published than studies suggesting equal or worse performance. However, in some cases there are legitimate reasons to exclude datasets that would be troublesome for the new method. If so, it should be clearly stated—understandable even to non-experts—that the inclusion criteria are defined so that the new method will achieve better performance and that the conclusions drawn from the benchmark study are then only valid for datasets satisfying these criteria. As with clinical trials, the inclusion criteria should not be tuned *a posteriori* to improve apparent performance of any given method. Finally, we point out that a conscious selection of promising datasets is not always sensible: it may indeed be interesting to also assess performance on datasets for which the new method does not perform as well in order to better delimit its appropriate field of application.

## Further EBM-related concepts

This section discusses further EBM-related concepts from clinical research and their possible adaptation to the context of computational statistical research; see Table 2 for an overview.

### Registration and protocols

In clinical trials, study protocols are written before the start of the trial to ensure the quality of the study and to fix decisions on some of the aforementioned issues, such as sample size, inclusion and exclusion criteria, study design, analysis methods, definitions of subgroups, the handling of missing values, methods to protect against bias and presentation and judgement of results. Deviations from the protocol decided at a later time or procedures that are followed but were not described in the protocol damage the quality of a study, even if not left unexplained. For example, a change of the primary endpoint, i.e. of the main research question focused on in a trial, draws the validity of findings into question. This is also a matter of concern in benchmark studies when several performance measures, such as estimates on the method’s prediction error, accuracy or computation time, are investigated. False-positive findings and biased results can be avoided when one of the measures is declared the primary endpoint before analysis. Registration of a trial in a registry for public availability [25] is another option to improve quality control of the clinical trial. Although information provided in this way is not as detailed as in a study protocol it is at least informative on the initial study goals.

More recently, the journal *Cortex* launched a new innovation in scientific publishing called a “Registered Report” [26]. The principle is to split the review process into two stages. Initially, experimental methods and proposed analyses are pre-registered and reviewed prior to data collection. Following peer review, positively evaluated proposals are offered “in-principle acceptance”. The authors then proceed to conduct the study while adhering to the registered report. After a second peer review by the same panel, the final manuscript is published regardless of the results, i.e. no matter whether the results are significant or not [26]. Since 2013 this concept has been adopted by various journals from different scientific fields.

Registration of a study and adherence to a protocol are concepts that may be partly transferable to benchmark studies and might help prevent or decrease both the fishing for significance problems [27] and publication bias [24]. The OpenML platform [4] provides these possibilities in the field of machine learning. Study registration or use of a protocol in computational sciences would, among other benefits, help to avoid the unjustified exclusion of methods or datasets from a benchmark study or the *a posteriori* fine-tuning of model parameters performed for presentation of favorable but biased results.

### Quality control

Quality control has been developed in EBM to prevent erroneous execution of a trial, faulty data generation and incomplete or incorrect reporting, where proper reporting also includes proper reporting of statistical analysis. For example, it is stated in the Guideline E6 of the International Council for Harmonisation of Technical Requirements for Pharmaceuticals for Human Use (ICH) that “the sponsor is responsible for implementing and maintaining quality assurance and quality control systems with written ’Standard Operating Procedures’ (SOPs) to ensure that trials are conducted and data are generated, documented (recorded), and reported in compliance with the protocol [ …]” and that “quality control should be applied to each stage of data handling to ensure that all data are reliable and have been processed correctly”. It is evident that the reliability of data and a quality controlled handling of data are not of minor importance in benchmark studies. “Quality control systems” such as monitoring and SOPs might be partly transferable to the context of benchmark studies, where the role of the sponsor may be taken over by a methodological researcher involved in the planning but not in the carrying out of the benchmark study (see Table 1). Again, exchange platforms such as OpenML [4] could be useful for this purpose.

### Placebos

A benchmark study conducted to compare the performances of two or more prediction models can be equated to a multi-arm clinical trial. When no standard therapy exists to which the new experimental treatment can be contrasted in a clinical trial, the trial is often placebo controlled. In the same manner, a benchmark study on the performance of competing prediction models could be “placebo controlled” by including in the comparison a method that, by design, performs no better than chance. For example, in a dataset where the outcome is a rare event with a prevalence of 5*%*, a reference method could be a naive classifier that always votes for the majority class, thus achieving a misclassification rate of only 5*%*. A useful prediction model would have to be able to outperform this reference method. In this specific example the necessary level of the performance of the reference method is obvious, but in more complex cases the control method would have to be designed carefully.

### Bias

#### Neutrality and blinding

According to Boulesteix et al. [19], a benchmark study can be considered *neutral* if (A) the main focus of the article is the comparison itself, which implies that the primary goal of the article is not to introduce a promising new method; (B) the authors are reasonably neutral; (C) the evaluation criteria, methods, and datasets should be chosen in a rational way. Requirement (B) means that the authors of a neutral comparison study do not have a preference for any particular method and, further, that they are (at least as a collective) approximately equally experienced with each of the considered methods. However, these requirements are very difficult to fulfill in practice, even if there exist positive exceptions; see for example the comparison study between splines and fractional polynomials [28] co-authored by experts of these two approaches.

In practice, researchers performing comparison studies often expect or hope for good results for a particular method—for example because they are those who developed it in previous research—thus perhaps consciously or unconsciously favoring it in a variety of possible ways. We conjecture that such mechanisms were at work in some of the studies discussed in “Motivating example” section, even if they were originally intended as neutral. In analogy to the biased assessment in the clinical context, researchers conducting benchmarking may select datasets or performance measures that are likely to yield good results for a “preferred” method. Similarly to differential patient care, researchers may perform parameter tuning more carefully, or fix bugs more eagerly, for a preferred method. Biases may also be a hazard even when a study is intended as neutral, i.e. does not aim to demonstrate the superiority of a particular method. And although it is well known that a new intervention must compete against the current standard of care in clinical trials, such a practice is not widespread in the context of comparison of statistical methods. Though it does not introduce bias in the strict sense, the selection of weak competitors in a benchmarking study might lead to an exaggeration of the superiority or advantages of, again, a preferred method. In a few words, failure to fulfill requirement (B) may produce bias.

To avoid this bias, strategies inspired from *blinding* for clinical trials might be imagined. In clinical settings, blinding (meaning that the patient and—in case of double-blinding—the caregivers and persons evaluating the endpoint are unaware of to which study group the patient belongs) is effective in helping to avoid several sources of bias in clinical trials. For example, to ensure unbiased assessment of a patient’s primary endpoint, a trial may be observer blind, i.e. endpoint assessment is performed by a person who does not know how the patients were treated. Medical staff can also be blinded to ensure no differential care of the patients. The feasibility of blinding strategies in the context of statistical research obviously depends on the characteristics of a specific study. In the rest of this section, we give first suggestions on how to use blinding strategies for improving neutrality in benchmarking studies.

We use the concept of “unexpectedly bad results” as an example. An unexpectedly bad result suggests that something went wrong with the considered method and that there may be an error somewhere. In analogy to blinding in clinical trials, we suggest that the handling of such problems might initially be conducted blindly, i.e. the decision to look for bugs or not should be taken without knowing which method yielded the unexpectedly bad result, in analogy to the blinding of medical staff. To implement this idea one might for example label the methods with non-informative names such as A, B, C, etc., withholding method-specific error messages from the debuggers and not unmasking the methods until the end of the study. Similarly, researchers could be blinded to the data through forbidding inspection of datasets that yield “unexpected results”, thus reducing the ability to exclude datasets—or fine-tune a method—in order to achieve better results *a posteriori*. Although one might perform the data inspection at a later point, the suggested blinding procedure would imply that this later inspection would not affect the reporting of the main benchmarking results.

More generally and beyond the strict concept of blinding, some decisions on study design, such as the selection of competing methods or performance measures, may be (partly) delegated to neutral persons if the principal investigators are not neutral themselves. The definition of strict inclusion criteria and the use of collaborative platforms such as OpenML [4] to systematically extract datasets satisfying these criteria, thus automatizing the benchmarking process, are also measures to counteract the effect of non-neutrality with regard to selection of datasets.

#### Non-compliance and missing values

In clinical trials, all enrolled patients are included in the statistical analysis: no patients are eliminated due to their outcome and the handling of patients who fail to comply to the treatment of the study arm in which they were included is delicate. More generally, the handling of missing values is considered crucial, and is given a great deal of attention in the literature. In particular, the intention-to-treat analysis strategy stipulates that all patients randomized in a clinical trial should be analyzed according to the initial randomization. Arbitrary exclusion from analysis or systematic drop-out can lead to severe bias.

In contrast to medical sciences, the issue of missing values has been given poor attention in the computational literature. Missing values may occur for different reasons in the context of benchmarking. For example, values may be missing “by design”, because a particular method was not applicable to a particular dataset. In this case, the benchmarking results should be analyzed and interpreted while taking these design issues into account. Missing values may also occur “accidentally”, because a method unexpectedly failed to produce a result for a dataset, for example, due to the non-convergence of the fitting process.

Similarly to the principle of intention-to-treat analyses in clinical research, all datasets originally included in a benchmark study would ideally remain for analysis, where appropriate treatment of these accidental missing values has to be defined depending on context. See Bischl et al. [29] for one of the few papers we are aware of explicitly stating how the missing values are handled. For example, if a method does not output a meaningful result for a considered dataset, one may set the performance of this method to the performance of the reference (when such a reference exists) or to the performance of the worst method (which makes sense only if more than two methods are compared). Post-hoc exclusion needs to be avoided and might only be acceptable if exclusion strategies are established beforehand and if based on decisions from a blinded review of results.

#### Role of the user

An important aspect we have not yet discussed is the role of the user in a benchmark experiment. In this paper, we have assumed that a method is well defined and runs automatically on the datasets without human intervention such as parameter initialization or preprocessing. This may hold true for some methods, such as a classification method without parameters or with parameters that can be efficiently tuned by cross-validation, but not for all. Similar issues may affect clinical trials. The counterpart of a method running without human intervention would be, for example, a drug that is produced and administered to the patients in a standardized way, so that the medical staff caring for the patient have no influence on the process. Obviously in the case of interventions in the form of, e.g., surgery, physiotherapy or psychotherapy, human factors can affect the quality of the intervention.

In the context of benchmarking for the evaluation of classification methods, Duin [30] distinguishes between studies comparing classifiers running without user intervention and studies comparing classifiers necessitating human intervention. In the latter case, Duin [30] again differentiates between benchmark experiments evaluating methods as used by a handful of expert users—experiments meant to yield the most reliable information on the method’s performance in optimal conditions, the only way to obtain a picture of the maximal potential of the method—and those involving “arbitrary users” (whereby the results of a method could be averaged over the users). In practice, however, this differentiation is rarely addressed in benchmarking literature. Complexities involving users could be seen as an argument against benchmarking in general, or in favor of extended benchmarking with the user considered as a factor. In the following we briefly sketch different scenarios regarding the expertise of the user in cases where methods require human intervention.

In the clinical context, it is obvious that the levels of expertise of the staffs conducting the compared interventions should be as equal as possible. For instance, if one intervention is conducted by experienced leading experts only and another intervention is conducted by young resident physicians only, the direct comparison will be biased. Similarly, in benchmarking, if an expert of method A uses both method A and a method B he/she is not familiar with, the comparison between methods A and B would be flawed. If there are no scientists with expertise in both methods A and B, a solution could be to involve several experts, each running only the method in which he/she is an expert. However, it is then impossible to distinguish between the effect of the method and the individual effect of the expert. To allow this distinction, the experts would have to also run the methods with which they are less familiar—an ethically controversial idea in clinical settings but possible in the context of benchmarking. In any case, the results of this type of benchmarking study should be seen as conditional on the involved experts. To eliminate this conditional character, one would have to draw a sample of experts, a difficult task in practice.

It may however also be interesting to get a picture of the performance of methods in standard settings, i.e. those involving arbitrary users without expertise in the considered methods. Although the results of the benchmarking study would be again conditional on the users, considering a sample of arbitrary users would address this issue. Finally, note that the performance difference between the methods that is estimated in this setting would not necessarily be equal to the performance difference when experts are involved, as methods do not necessarily suffer equally from the lack of experience of their users.

These ideas are certainly difficult to implement in practice, but potential precedence exists. In the machine learning field initiatives such as the aforementioned collaborative platform OpenML [4], which collects and stores the results of benchmarking experiments performed by a large number of researchers featuring users of various levels of expertise, may provide a technical framework to address user-related issues.

### Levels of evidence

**Fig. 1:**
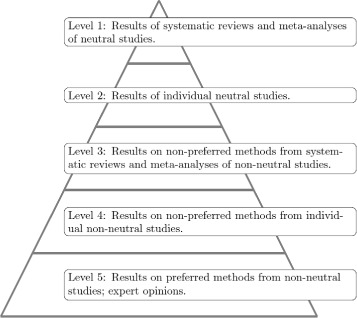
Evidence pyramid. Suggested levels of evidence for results of benchmark studies designed for the comparison of statistical methods using real data. A neutral study is conducted by researchers that do not have a preference for any particular method and are (at least as a collective) approximately equally experienced with each of the considered methods. A non-neutral study is one in which the researchers have a potential conscious or subconscious interest in the demonstration of the superiority of a given method (the “preferred method”) or have greater experience in one or more of the methods (again, the “preferred method”) to the extent that it may bias the results. A non-preferred method is a statistical method from a non-neutral study but not that or those method(s) thought to be preferred. Bias in non-neutral studies can advantage preferred methods and disadvantage non-preferred methods, or both

Suggestions on “levels of evidence” have been made in the literature to aid in the assessment of the quality of information derived from clinical studies; see the report of the Canadian Task Force on the Periodic Health Examination for an early seminal work [31]. These suggestions present a rough rating scale in which systematic reviews and meta-analyses provide the highest level of evidence, followed by high quality randomized controlled trials, cohort studies, case-control studies and finally, expert opinions. The quality of benchmark studies could be, in principle, rated in a similar way. Figure 1 suggests ideas for a possible classification system. Much of the system is shaped by the concept of neutrality, whether a paper is introducing a new statistical method or there is otherwise a “preferred” method, whether consciously or subconsciously.

As stated earlier, when a comparison study is presented in a paper that introduces a new method, results tend to be biased in favor of the new proposal [32]; moreover, the competing methods often show comparatively bad performance, as they have consciously or unconsciously been put in an inferior position (e.g., through suboptimal parameter settings or failure to fix bugs). As such, results related to the introduction of new methods rank consistently lower on the hierarchy. Similarly, when neutrality is affected through differential expertise in the examined methods, the quality of the results again suffers. In general, bias in non-neutral studies can advantage preferred methods and disadvantage non-preferred methods, or both.

Starting at the highest level, like on the EBM scale, meta-analyses and systematic reviews occupy the highest position, although in the context of computational science these studies are still in their infancy and not straightforward methodologically [33, 34]. Specifically, to achieve the highest level of evidence, these meta-analyses and systematic reviews must be based on high quality *neutral comparison studies* [19]. The neutral comparison studies themselves are considered the second highest level of evidence on the scale, followed by systematic reviews and meta-analyses of non-neutral comparison studies, but only those that exclude the results of the individual newly introduced methods or the otherwise preferred method. Finally, results on the non-preferred methods from these individual non-neutral studies are of the second lowest level of evidence, followed by expert opinion and results from non-neutral studies on preferred methods.

## Conclusion

The appropriate design of clinical trials has been the subject of decades of research, the goal of which has been to improve the quality and reliability of research findings. We have described an analogy to this evidence-based medicine in the field of methodological computational statistics. We suggest that benchmark studies—a method of assessment of statistical methods using real-world datasets—may benefit from adopting concepts from EBM towards the goal of more evidence-based statistical research. In particular, we have discussed the application of inclusion and exclusion criteria to the selection of datasets, and the use of placebos, study protocols and methods of protecting against bias as common concepts in the clinical world which would be beneficial in the design and interpretation of high-quality benchmark studies.

We applied some of these ideas in a recent benchmark study comparing random forest and logistic regression currently available as a technical report [35]. In this study, we perform sample size calculations, clearly define the set of candidate datasets and the inclusion criteria, and report missing values transparently. Moreover, the authors of the study are equally familiar with both considered methods and have conducted research projects on both of them in the past, thus ensuring a high level of neutrality.

The questions of whether the choice of statistical methods for real data analysis should be based on evidence that is itself based on real-data and how this could be achieved can obviously not be answered by single authors. Our manuscript is meant to fuel discussion on the paradigms and challenges faced in computational statistics and suggests first potential steps towards more “evidence-based” statistical research rather than offering clear-cut guidance. However, we feel that the definition of inclusion criteria for datasets, a clear statement on the handling of missing values and a careful and fair-minded consideration of issues related to the researcher’s varying levels of competence regarding the compared methods should currently be the minimal requirements for evidence-based statistical research—requirements easily satisfied in practice and without hidden danger.

## Abbreviations

EBM: Evidence-based medicine
STRATOS: STRengthening analytical thinking for observational studies
